# Laparoscopic Approach to Rectal Cancer—The New Standard?

**DOI:** 10.3389/fonc.2020.01239

**Published:** 2020-07-31

**Authors:** Christine Hsieh, Kyle G. Cologne

**Affiliations:** Keck School of Medicine, University of Southern California, Los Angeles, CA, United States

**Keywords:** rectal cancer, laparoscopy, colorectal cancer, robotics, controversies

## Abstract

Minimally invasive surgery has revolutionized the way surgeons perform colorectal surgery, and new technologies continually upend the way surgeons view and operate within the deep pelvis. Among other benefits, it is associated with decreased lengths of stay, wound and surgical site infections, pain scores, and has an overall lower complication rate vs. open surgery (1). Recently, however, the role of minimally invasive surgery has been called into question in the effective and safe treatment of rectal cancer. This manuscript will outline the history of minimally invasive rectal cancer surgery, examine evidence detailing its safety (compared with alternatives), and discuss important aspects of use, most notably the considerable learning curve required to achieve proficiency, the extent of its current use, and potential pitfalls. The current evidence suggests minimally invasive surgery is a very safe way to treat rectal cancer when performed by experienced and specialty trained surgeons.

## History

Laparoscopy was described by Aulus Cornelius Celsus (25 B.C.−50 A.D.), who used percutaneous devices (now called trocars) to “drain evil humors” (2). A number of advances made laparoscopic procedures possible. In 1929, Heinz Kalk, a German gastroenterologist, developed an angled lens system and a dual trocar approach. Ten years later, he published this experience including 2,000 laparoscopic liver biopsies under local anesthesia without a single mortality (3).

However, early laparoscopy was dangerous. There was a high rate of trocar injury to bowel, only unipolar cautery (which caused a number of additional bowel and other organ injuries), poor lighting, and no good way to regulate insufflation pressures. Finally, in 1952, Fourestier et al. (4) developed a new lighting system using a quartz rod to transmit an intense light beam distally along a telescope. Light could finally be transmitted with enough intensity to be concentrated and allow photographed images (4). In 1960, German gynecologist Kurst Semm invented an automatic insufflator (5). Finally, in the early 1980s, the first solid-state camera was introduced that allowed video-laparoscopy (whereas previously an eyepiece was required that only allowed a single observer to see through the scope).

Despite these advances, surgeons were reluctant to perform more complex laparoscopic surgeries in part due to early dangers. Mühe performed the first laparoscopic cholecystectomy in the mid-1980s (6, 7). Development of the laparoscopic stapler allowed the first colorectal procedures to be performed, which included a right hemicolectomy performed by Moises Jacobs in June of 1990; Dennis Fowler performing a sigmoid resection in October of 1990. In November 1990, Joseph Uddo used a circular stapling device to perform a laparoscopic colostomy closure, and Patrick Leahy resected a proximal rectal cancer with low anterior anastomosis (8, 9). After reports of the success of these initial colorectal procedures, surgeons began to more frequently employ laparoscopic surgery for colorectal cancer.

## Evidence of Oncologic Safety

Early reports of trocar site recurrences following laparoscopic resections raised alarm and were associated with calls for a moratorium on laparoscopic colorectal cancer surgery (10). In addition, early studies of laparoscopic treatment of rectal cancer showed a trend toward higher rates of positive circumferential margins and a high conversion rate (34%) (11). It was presumed that some of these poor results were due to procedures performed during the learning curve phase before mastery, as only 20 previous case experiences were required to enroll in these trials. It soon proved that abdominal wall recurrence rates were no different than open approaches.

To better investigate the issue of laparoscopic safety in general, several large randomized trials were designed and carried out. Initial results with long-term follow-up showed no difference in survival and local recurrence rates when comparing laparoscopic to open approaches. In fact, laparoscopic approaches showed some advantages over open surgery. The COST trial (12), COLOR I and II trials (13, 14), CLASICC trial (11, 15), and COREAN (16) demonstrated non-inferior outcomes to open surgery (Table 1). Potential short-term benefits of the laparoscopic approach were also discovered in a Cochrane Review, where laparoscopic approach resulted in the same improvements that were noted previously (decreased blood loss, quicker oral intake, decreased narcotic use, and lower rates of surgical site infections) (18). Furthermore, Arezzo et al. performed a meta-analysis in 2013 of prospective trials evaluating laparoscopic vs. open rectal resection involving 23 studies (eight randomized) and 4,539 patients. They found a mortality reduction of 2.4 1.0% favoring the laparoscopic group (*p* = 0.048) and decreased morbidity (35.4 vs. 31.8%, *p* < 0.001) as well (19).

**Table 1:** Long-term outcomes of randomized controlled trials comparing minimally invasive to open approaches for rectal cancer.

**Study**	**Disease-free survival (lap vs. open)**	**Local recurrence (lap vs. open)**	**Distant recurrence (lap vs. open)**
COLOR II (14)	74.8 vs. 70.8%	5 vs. 5% (4.4 vs. 11.7% in low cancers)	
COREAN (16)	79 vs. 72%	2.6 vs. 4.9%	
Z6051 (17)	79.5 vs. 83.2%	4.6 vs. 4.5%	14.6 vs. 16.7%

However, in 2015, two randomized trials were published that questioned the safety of minimally invasive rectal cancer surgery. ALaCaRT and Z6051 (20, 21) used a composite score which included circumferential resection margin, distal margin, and completeness of the total mesorectal excision (TME) specimen as a short-term quality metric. TME refers to the complete removal of the rectum and its mesentery along anatomic and fascial planes. All three areas were required to be negative or complete/nearly complete to achieve the composite definition. With a combined 961 patients, the authors in both studies failed to show non-inferiority of the laparoscopic approach. While the absolute differences were not statistically significant between each arm, the 95% confidence interval for this composite score crossed the predetermined definition of non-inferiority (of 6%). Therefore, the authors concluded that the laparoscopic approach could not be deemed non-inferior.

This created an uproar and a lot of confusion after release. Entire symposia at national meetings were devoted to this topic. The criticism of these studies was that the conclusions were based on short-term metrics, which were a surrogate for the true areas of interest included in other randomized studies: local and distant recurrence rates. While these were randomized studies, other authors have previously looked at short-term pathologic outcomes and found different results (suggesting short-term pathologic outcomes were equivalent): Creavin et al. (22) performed a meta-analysis of four studies and 2,319 patients. They found that while superficial mesorectal defects were more common in laparoscopic approach, no differences were seen in acceptable rates (complete + nearly complete) of mesorectal grade, circumferential margin (CRM) positivity, and distance to radial and distal margins between laparoscopic and open groups. They therefore concluded that these differences have not affected oncological outcomes. In another meta-analysis, Milone et al. (23) also found no statistically significant differences between the two groups among 12 trials. Sara et al. (24) found no difference in CRM positivity and the number of lymph nodes between laparoscopic and open approaches. Boutros et al. (25) found no difference in CRM, distal resection margin, and completeness of TME specimen. Penninckx et al. (26) also showed no difference in TME specimen quality, CRM positivity, and number of lymph nodes. De Jesus et al. (27) performed an analysis of laparoscopic, robotic, and open procedures and also found no difference in circumferential margin status by approach.

Since that time, the authors of the ALaCaRT and Z6051 studies have published longer term follow-up (Table 1) (17) that showed no difference in 2-year disease-free survival or locoregional recurrence rates. Similarly, a number of other studies have demonstrated no difference in laparoscopic vs. open surgery outcomes with longer term follow-up variables of local recurrence and disease-free survival (28–35). As a result, minimally invasive surgery is accepted as at least as oncologically effective as open surgery. Furthermore, minimally invasive approaches have been associated with short-term benefits such as reducing length of stay, blood loss, and pain. While this may be confounded by the adoption of enhanced recovery protocols, there is gathering evidence that laparoscopy directly impacts these factors (36).

## Learning Curve

Laparoscopic rectal resection is in every sense of the word complex. It requires surgery in multiple quadrants (to mobilize the splenic flexure and dissect in the low pelvis), large vessel ligation, bowel transection, and reanastomosis. These tasks require a significant degree of skill particularly because multiple specialized (open) retractors are not available, and the surgeon must interpret a three-dimensional environment on a two-dimensional screen. This can be a daunting task. Proper identification of planes that may be distorted by radiotherapy is also key to performing effective surgery with good oncologic results. Finally, medial to lateral dissection is not often performed in open procedures, so identifying these planes requires a different skill set than traditional open surgery. Thus, laparoscopic procedures often take longer than open ones and are not for the faint hearted or easily frustrated surgeon. In reference to these complex laparoscopic cases, Theodore Saclarides once said: “The patient looks better than the surgeon the next day.” Anyone who has performed laparoscopic ultralow anterior resection in an obese male can relate to this statement.

Many of the aforementioned randomized trials on outcomes for laparoscopic surgery required surgeons to demonstrate successful performance of 20 procedures, as this was initially considered to be the learning curve (12). It became clear during the trials that this was an underestimation, as outcomes improved in the later part of the studies. A subsequent investigation using a cumulative sum method (and adjustment for case mix index) demonstrated that 55 procedures were the learning curve for right colectomy and 62 procedures for left colectomy (37). Specialized training programs in colorectal surgery may allow more rapid ascent of this learning curve. Indeed, higher surgical volume has been correlated with lower complication rates and reinterventions for early to locally advanced rectal tumors, while fewer R0 resections of T4 tumors were achieved in lower volume hospitals (38). Conversion rates (a surrogate marker for surgeon expertise) have declined over time. Risk factors for conversion have been well documented and include surgeon experience, obesity, male gender, and higher ASA score (39). Other risk factors are related to the characteristics of the tumor itself (40) and any operative complications such as bleeding or injury to other structures, or even equipment difficulties. It is for this and many other reasons that the National Accreditation Program for Rectal Cancer (NAPRC) exists—to ensure quality outcomes and expertise in treating this disease process.

Technology has also aided progress. At the time of these trials, high-definition video laparoscopes were not readily available. Advanced Energy devices are now several generations further in evolution as well and include the Harmonic® ACE (Ethicon Endo-Surgery, USA), LigaSure™ (Covidien, USA), and EnSeal® (Ethicon) among others, giving the surgeon greater flexibility to transect vessels varying from 5 to 7 mm in size (41), which may have previously required stapling. Finally, reticulating staplers themselves have better technology that allows transection of bowel deeper within the pelvis (42).

## Pushing the Boundaries of Current Technology: Robotics and More

As surgeons adapt laparoscopy to increasingly complex situations, the limitations of this technique become increasingly clear. This is no more evident than in rectal cancer surgery. The laparoscopic camera allows for enhanced visualization and magnification in the deepest recesses of the pelvis, and some advanced flexible laparoscopes offer 3D optics while maintaining a low enough profile to accommodate the working instruments. Still, despite improved visualization, access to these tight spaces is still challenging with the usual straight instruments which must work around the fulcrum of the instrument trocar. Some wristed laparoscopic instruments are available and are designed to facilitate delicate tasks like suturing and intracorporeal knot-tying. As an added expense, these are not yet widely used and, in many cases, require considerable practice in order to use smoothly.

These limitations, in addition to the significant maneuvering and stamina required of the surgeon during these challenging cases, make the robotic platform increasingly attractive for use in the deep pelvis. There is no denying the appeal of stable, articulating instruments that mimic the motion of the surgeon's hands in the restrictive bony confines of the pelvis while allowing the operator to remain comfortably seated and focused with 3D visualization on the field. However, the drawbacks are considerable. Using a camera trained onto the operative field can reduce contextual clues and landmarks taken for granted in open surgery. The ability to move easily from one quadrant to another can be hampered as well. And certainly, tactile feedback is drastically altered in laparoscopy compared to open surgery and eliminated altogether in robotic surgery. But given how quickly the field of robotic surgery is developing, it seems that it is only a matter of time before these disadvantages are lessened, if not eliminated completely.

The first step in evaluating robotic technologies is to compare outcomes to laparoscopic surgery. Early outcomes are promising. In an analysis of the National Cancer Database, robotic low anterior resection showed a similar length of stay, readmission rate, and 30-day mortality compared to laparoscopy. When assessed in aggregate, the minimally invasive approach was associated with shorter length of stay, otherwise the abovementioned parameters were comparable to the open approach (43).

Conversion rates are another area to examine, particularly as previous trials have described a high conversion rate in the low pelvis in performing cancer surgery. While studies emphasize lower rates of conversion compared to the laparoscopic approach (44), whether or not lower conversion rates translate to better outcomes rather than represent qualities inherent to the patient and their cancer remains to be seen for minimally invasive approaches in general.

Data regarding bowel, urinary, and sexual function tend to report the robotic approach as equivalent (45), if not improved. Kang et al. (46) performed a case-matched study of open, laparoscopic, and robotic approaches and found that the patients in the robotic arm tolerated diet faster and had a shorter length of stay compared to the laparoscopic group. They also demonstrated lower postoperative pain scores, less voiding dysfunction, and lower CRM positivity in the robotic group compared to the open surgery group. There was, however, no significant difference in disease-free survival at 2 years when assessing robotic, laparoscopic, and open groups (46). In a prospective study of patients undergoing laparoscopic or robotic TME for rectal cancer, the authors found that quality of life was comparable between robotic and laparoscopic approaches; however, there seemed to be less urinary and sexual dysfunction in the robotic group (47). Though many studies exist, they are often small single-institution studies underpowered to definitively answer these questions. Most, however, show equivalence or slight advantages over laparoscopic approaches. Just with comparing laparoscopic to open approaches, an additional area to examine in the laparoscopic vs. robotic arena is the quality of the pathologic specimen. In a recent randomized controlled trial (RCT) comparing laparoscopy to robotic rectal cancer surgery, Kim et al. (48) demonstrated comparable TME quality, with no statistically significant difference in margins, morbidity, and bowel function compared to laparoscopic resection. Oncologic outcomes in many single-institution studies are in agreement regarding equivalent short-term overall survival and disease-free survival (49–51).

There are increasingly more studies examining longer term outcomes. Park et al. published their 5-year overall survival rate, disease-free survival, and local recurrence rates, showing similar results for the laparoscopy and robotic surgery groups. The authors did however note that the patient's payment for robotic surgery was more than twice as much as for laparoscopic surgery (52).

Currently, there are no compelling long-term data that make robotic rectal cancer surgery clearly advantageous over open or laparoscopic approaches. The ROLARR trial attempted to establish this, randomizing 471 patients to robotic or laparoscopic resections of rectal cancer and involving 40 surgeons from 29 sites in 10 different countries to achieve this end. The ROLARR trial results showed that there was no statistically significant difference in odds of conversion to open when comparing robotic-assisted to laparoscopic surgery. However, subset analysis suggested that there was more conversion to open in obese patients undergoing laparoscopic rather than robotic surgery. Conversion rates were noted to be elevated in subgroup analyses of males and those with low tumors and were as high as 18.9% for obese patients. The oncologic results were equivocal (53).

As an elaboration of the minimally invasive approach, robotic surgery's short-term benefits so far seem to track accordingly. However, there are still considerable hurdles regarding cost-effectiveness, accessibility, and adequate training which must be addressed. Furthermore, clear evidence of long-term oncologic benefit must be established. While the rapidly building body of literature supporting robotic surgery suggests comparable or even favorable outcomes using the robotic platform, one must bear in mind the inherent bias in these publications. Scores of retrospective studies are suggestive, but certainly not definitive, and it is of course not possible to “blind” the healthcare providers at all patient touchpoints to the intended treatment approach. Furthermore, the dividing line between significance and non-significance can be increasingly manipulated. An eyebrow-raising analysis of 38 studies comparing robotic colorectal surgery to other approaches found that “spin”—defined as “specific reporting that could distort the interpretation of results and mislead readers”—affected over 80% of the included studies (54).

In the meantime, novel techniques such as transanal TME are being studied as a potential means of overcoming the limits of laparoscopic surgery. For now, the robust efficacy and oncologic data, plus the clear short-term benefits to patients of the laparoscopic approach, make this the most feasible and beneficial technique for rectal cancer surgery today.

## Transanal Total Mesorectal Excision

In addition to robotic approaches, a novel technique has the potential to redefine minimally invasive rectal cancer surgery. Transanal total mesorectal excision (TaTME) is a novel approach which seeks to overcome the challenges which hamper both laparoscopic and robotic approaches to low rectal tumors. Early postoperative outcomes are encouraging, showing no significant difference in 30-day postoperative complications (55) and oncologic outcomes which are equivalent (56) or even improved compared to laparoscopy (57). As with any innovation, there have been some hurdles with this approach, such as the troubling frequency of carbon dioxide embolus (58) and urethral injury (59, 60). Long-term results are not yet available, but they are being systematically gathered thanks to several international registries and ongoing trials, such as the COLOR III trial currently enrolling patients to assess laparoscopic TME compared to transanal TME. Currently, there is not enough evidence to routinely recommend this approach, particularly given the reported high morbidity and high learning curve (61, 62).

## Conclusion

Overall, minimally invasive approaches to the treatment of rectal cancer have a growing track record for providing safe treatment. These procedures are not easy to perform and do have a steep learning curve. But when performed by trained surgeons, they have numerous advantages and can be considered the preferred approach (when possible) for the treatment of rectal cancer, including locally advanced tumors that require neoadjuvant chemoradiotherapy.

## Author Contributions

All authors listed have made a substantial, direct and intellectual contribution to the work, and approved it for publication.

## Conflict of Interest

The authors declare that the research was conducted in the absence of any commercial or financial relationships that could be construed as a potential conflict of interest.
